# Use of analgesics in intentional drug overdose presentations to hospital before and after the withdrawal of distalgesic from the Irish market

**DOI:** 10.1186/1472-6904-10-6

**Published:** 2010-03-18

**Authors:** Paul Corcoran, Udo Reulbach, Helen S Keeley, Ivan J Perry, Keith Hawton, Ella Arensman

**Affiliations:** 1 National Suicide Research Foundation, 1 Perrott Avenue, College Road, Cork, Ireland; 2 Department of Psychiatry, School of Medicine, University of Oviedo, Centro de Investigación Biomédica en Red de Salud Mental (CIBERSAM), Julian Claveria 6, 33006 Oviedo, Spain; 3 Department of Epidemiology and Public Health, University College, Cork, Ireland; 4 Child and Adolescent Mental Health Services, North Cork Area, Health Service Executive South, Mallow, County Cork, Ireland; 5 Centre for Suicide Research, University of Oxford, Department of Psychiatry, Warneford Hospital, Headington, Oxford OX3 7JX, UK

## Abstract

**Background:**

Distalgesic, the prescription-only analgesic compound of paracetamol (325 mg) and dextropropoxyphene (32.5 mg) known as co-proxamol in the UK, was withdrawn from the Irish market as of January 2006. This study aimed to evaluate the impact of the withdrawal of distalgesic in terms of intentional drug overdose (IDO) presentations to hospital emergency departments (EDs) nationally.

**Methods:**

A total of 42,849 IDO presentations to 37 of the 40 hospitals EDs operating in Ireland in 2003-2008 were recorded according to standardised procedures. Data on sales of paracetamol-containing drugs to retail pharmacies for the period 1998-2008 were obtained from IMS Health.

**Results:**

The withdrawal of distalgesic from the Irish market resulted in an immediate reduction in sales to retail pharmacies from 40 million tablets in 2005 to 500,000 tablets in 2006 while there was a 48% increase in sales of other prescription compound analgesics. The rate of IDO presentations to hospital involving distalgesic in 2006-2008 was 84% lower than in the three years before it was withdrawn (10.0 per 100,000). There was a 44% increase in the rate of IDO presentations involving other prescription compound analgesics but the magnitude of this rate increase was five times smaller than the magnitude of the decrease in distalgesic-related IDO presentations. There was a decreasing trend in the rate of presentations involving any paracetamol-containing drug that began in the years before the distalgesic withdrawal.

**Conclusions:**

The withdrawal of distalgesic has had positive benefits in terms of IDO presentations to hospital in Ireland and provides evidence supporting the restriction of availability of means as a prevention strategy for suicidal behaviour.

## Background

Distalgesic (known as co-proxamol in the UK) is a prescription-only analgesic compound of paracetamol (325 mg) and dextropropoxyphene (32.5 mg). Evidence of the dangers of distalgesic in overdose began with a case-series study in Northern Ireland over 30 years ago [1] and culminated with a study that showed that the drug was the second most commonly used drug in overdose suicides in England and Wales, accounting for 5% of all suicides and with a higher risk of fatal outcome in overdose acts than other commonly used medicines [2]. Death from distalgesic overdose may occur rapidly, even with a relatively low dose, and lethality is increased by use of alcohol and other central nervous system depressants. As a consequence, the majority of overdose deaths occur before hospital treatment can be received [3]. After extensive review of the risks and benefit, the drug was withdrawn from the UK market over a three-year period, 2005-2007. Distalgesic was withdrawn from the Irish market as of January 2006.

The efficacy of various suicide prevention strategies to reduce the risk of suicide involving distalgesic has been evaluated, including educational strategies for doctors, restricting the number of prescribed tablets, restrictions on prescribing and complete withdrawal [3]. So far, restricting availability of distalgesic has produced positive results in the UK, Scandinavia and Australia [4-8].

This study aimed to evaluate the impact of the withdrawal of distalgesic from the Irish market in terms of intentional drug overdose (IDO) presentations to hospital emergency departments (EDs) nationally. Specifically, we compared the rate of presentations to hospital of IDO acts involving distalgesic before (2003-2005) and after (2006-2008) its withdrawal, tested for evidence of substitution whereby there was a change in the rate of overdose presentations involving other paracetamol-containing drugs and examined whether there was a change in the number of tablets taken in overdose presentations involving distalgesic. We also examined changes in sales to retail pharmacies of paracetamol-containing analgesics.

## Methods

### Data sources

The National Registry of Deliberate Self Harm collects data from all hospital emergency departments (EDs) in Ireland using an internationally-recognised definition that includes all non-fatal intentional drug overdose (IDO) acts [9]. Registry data are collected by trained data registration officers who operate independently of the hospitals and follow standard operating procedures. Quality control measures include regular team meetings to reinforce the standardised application of case-definition and ascertainment criteria as well as cross-checking exercises which have shown high levels of agreement and reliability across registration officers. A minimal dataset is collected, described in detail in the Registry's annual reports [10]. The dataset includes the age, gender and area of residence of the patient, data regarding the nature of the self-harming behaviour and whether the patient was hospitalised. If multiple IDO presentations are made by the same individual, all are recorded by the Registry. Ethical approval for the Registry was granted by the National Ethics Committee of the Faculty of Public Health Medicine as well as by local ethics committees.

Population data from the 2006 national census and annual population estimates for 2003, 2004, 2005, 2007 and 2008 (disaggregated by sex and age group) were obtained from the website of the Irish Central Statistics Office (CSO; http://www.cso.ie/). IMS Health supplied data on wholesale sales of paracetamol-containing drugs to retail pharmacies in Ireland for the period 1998-2008.

### Data analysis

Of the 40 hospital EDs operating in Ireland in 2003-2008, the Registry obtained data from 37 hospitals in 2003, 38 hospitals in 2004-2005 and all 40 hospitals in 2006-2008. Statistical analysis was limited to the data collected from the 37 hospitals that contributed to the Registry for the complete study period (2003-2008). These hospitals accounted for 86.5% of all Registry-recorded IDO presentations in 2006-2008. This proportion was used to derive a weighting (100/86.5 = 1.16) that was applied to the data of the 37 hospitals for all study years and these weighted data were used to calculate national presentation rates. Total, male and female rates of IDO presentations to hospital per 100,000 population were age-standardised using the European standard population [11]. Five year age groups (0-4, 5-9, ..., 80-84 and 85+) were used for the age-standardisation but when the data were more limited broader age groups were used (0-14, 15-34, 35-54, 55+). Assuming that the number of presentations (x) followed a Poisson distribution, 95% confidence intervals (CIs) for the rates were calculated using the Normal approximation, i.e. confidence interval = (x +/- 2*√x) * 100,000/population.

Data analysis was carried out relating to all IDO presentations, presentations involving any paracetamol-containing drug and presentations involving distalgesic. We also examined presentations involving the other common, paracetamol compound, prescription-only, moderate-severe painkillers that were available in the country across the study period (Kapake, Paramol, Solpadol, Syndol and Tylex; referred to as 'other prescription compound analgesics' in the paper) and presentations involving solpadeine, by far the most common over-the-counter paracetamol compound analgesic available only from pharmacies.

Chi-square tests were used to assess whether the proportion of IDO presentations to hospital involving distalgesic varied by sex, age and year. Poisson regression analysis was used to assess rate differences by sex and year and changes between the three pre-withdrawal years and the three post-withdrawal years collectively and separately (i.e. 2003-2005 vs. 2006-2008 and 2003-2005 vs. 2006, 2007, 2008). The number of distalgesic tablets taken was recorded in 84% of the IDO presentations involving the drug. Because of its skewed distribution, the number of tablets was summarised by the median and interquartile range. The non-parametric Mann-Whitney test was used to assess variation in the number of tablets taken between two groups and the Kruskal-Wallis test was used to compare more than two groups.

The statistical analysis was carried out using SPSS version 16.0 (SPSS Inc., Illinois, USA) except for the Poisson regression analyses, which were carried out using Stata version 6.0 (StataCorp, Texas, USA).

## Results

In 2003-2008, there were 42,849 recorded intentional drug overdose (IDO) presentations to the 37 hospital EDs that contributed fully to the Registry during these six years (annual mean (range): 7,142 (6,642-7,692)). Women accounted for 61.2% of all IDO presentations, a proportion that did not vary by year (Chi-square = 5.33, df = 5, p = 0.139). In 2,050 (4.8%) of the IDO presentations, it was not known what drugs were taken. The total, male and female annual rates of IDO presentations to hospital EDs were 188.4 (95% CI = 186.6-190.1), 143.5 (95% CI = 141.3-145.7) and 234.3 (95% CI = 231.5-237.0) per 100,000 population, respectively. Female sex was associated with a 60% higher rate of IDO presentations after adjustment for age (IRR = 1.60, 95% CI = 1.57-1.63). At least one paracetamol-containing drug was involved in 13,066 (30.5%) of the IDO presentations. The total, male and female annual rates of IDO presentations to hospital involving paracetamol were 57.0 (95% CI = 56.0-57.9), 35.4 (95% CI = 34.3-36.5) and 79.1 (95% CI = 77.5-80.7) per 100,000 population, respectively. After adjustment for age, female sex was associated with a more than doubling of the rate of paracetamol-related IDO presentations (IRR = 2.21, 95% CI = 2.13-2.28).

**Figure 1:**
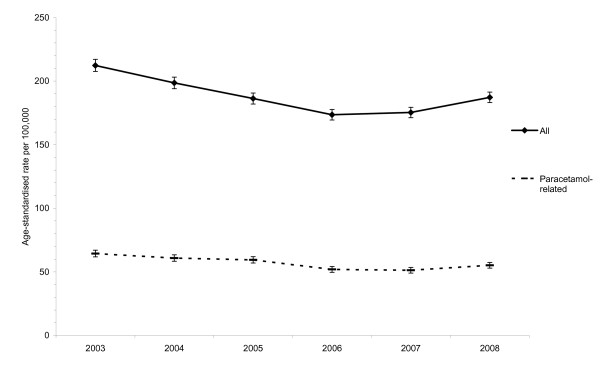
**National rate of intentional drug overdose presentations (all and those involving a paracetamol-containing drug) to hospital in Ireland, 2003-2008**. Note: Error bars represent the 95% confidence intervals for the rates.

The national rate of IDO presentations to hospital deceased over the study period (Figure 1). The rate was 212.3 per 100,000 in 2003 and fell annually by 6.7% (95% CI = 5.8-7.6%) to 173.6 in 2006. Between 2007 and 2008, the rate increased from 175.3 to 187.2 per 100,000, an increase of 7.0% (95% CI = 3.8-10.3%) after adjustment for age. This trend was also evident for IDO presentations involving a paracetamol-containing drug. The rate was 64.3 per 100,000 in 2003 and also fell annually by 6.7% (95% CI = 5.1-8.3%) to 51.9 in 2006. Between 2007 and 2008, the rate increased from 51.2 to 55.1 per 100,000, an increase of 6.5% (95% CI = 0.6-12.6%) after adjustment for age.

Sales to retail pharmacies of distalgesic, other prescription compound analgesics, solpadeine and other paracetamol-containing drugs increased 4-6% per year over the eight years (1998-2005) before distalgesic was withdrawn from the Irish market 2. In 2005, approximately 40 million tablets of distalgesic were sold to pharmacies. This fell to 500,000 in 2006, to approximately 2,000 in 2007 and to none in 2008. Between 2005 and 2006, there was a 48% jump in sales of the other prescription compound analgesics, an 11% increase in sales of solpadeine and a 22% increase in sales of other paracetamol-containing medicines.

**Figure 2:**
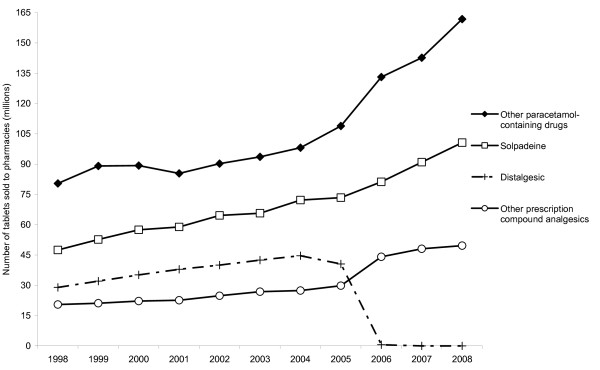
**Trends in sales to retail pharmacies in Ireland of paracetamol-containing medicines, 1998-2008**.

Of the 42,849 recorded IDO presentations, distalgesic was one of the drugs taken in 1,312 (3.1%) acts (Table 1). The involvement of distalgesic in IDO acts varied by sex (Chi-square = 12.13, df = 1, p < 0.001), age (Chi-square = 37.64, df = 4, p < 0.001) and year (Chi-square = 635.38, df = 5, p < 0.001). Distalgesic was more common in female IDO acts and was less common with increasing age. In the three years pre-withdrawal, it was involved in almost 400 presentations annually, approximately 5% of all IDO presentations. The three years post-withdrawal saw sharp reductions in the numbers and proportions of IDO presentations involving distalgesic (Table 1). A similar pattern was observed when distalgesic was considered as a proportion of all paracetamol-related IDO presentations. The drug was involved in 16% of paracetamol-related acts in 2003-2005 but this declined sharply in line with the distalgesic withdrawal (Table 1**)**.

**Table 1:** Intentional drug overdose presentations to hospital in Ireland involving distalgesic

		n	% of all IDO presentations	% of paracetamol-related IDO presentations
Sex	Male	448	2.7%	10.9%
	Female	864	3.3%	9.7%

Age	<15 years	42	4.7%	9.5%
	15-24 years	488	3.6%	8.9%
	25-44 years	556	2.8%	10.6%
	45-64 years	213	2.7%	12.4%
	65 years+	13	1.6%	8.3%

Year	2003	386	5.0%	16.2%
	2004	356	4.9%	15.7%
	2005	371	5.3%	16.5%
	2006	109	1.6%	5.5%
	2007	64	0.9%	3.2%
	2008	26	0.4%	1.2%

Total		1312	3.1%	10.0%

**Figure 3:**
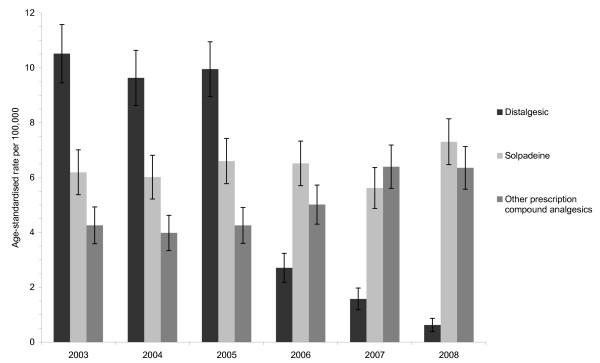
**National rate of intentional drug overdose presentations to hospital in Ireland involving distalgesic, other prescription compound analgesics and solpadeine, 2003-2008**. Note: Error bars represent the 95% confidence intervals for the rates.

The age-standardised rate of IDO presentations to hospital involving distalgesic was 10 per 100,000 in 2003-2005 (Figure 3). The rate fell in each of the three post-withdrawal years - to 2.7 (95% CI = 2.2-3.2) per 100,000 in 2006, 1.6 (95% CI = 1.2-2.0) per 100,000 in 2007 and 0.6 (95% CI = 0.4-0.9) per 100,000 in 2008. In contrast, the rate of IDO presentations involving the other prescription compound analgesics was approximately 4 per 100,000 in 2003-2005, rising to 5.0 (95% CI = 4.3-5.7), 6.4 (95% CI = 5.6-7.2) and 6.4 (95% CI = 5.6-7.1) per 100,000 in 2006, 2007 and 2008, respectively. There was no trend evident in the rate of IDO presentations involving solpadeine.

The rate of IDO presentations to hospital involving distalgesic in 2006-2008 was 83.5% lower than what it had been in the three years before it was withdrawn (Table 2). This decrease was graded across the three years. Compared to 2003-2005, the rate of distalgesic-related IDO presentations was 72.3% (95% CI = 66.7-76.9%) lower in 2006, 84.0% (95% CI = 79.8-87.3%) lower in 2007 and 93.8% (95% CI = 91.0-95.7%) lower in 2008. The rate of IDO presentations involving the other prescription compound analgesics was 43.5% higher in 2006-2008 than in 2003-2005. The increase were somewhat graded - 22.0% (95% CI = 4.4-42.5%) higher in 2006, 54.5% (95% CI = 34.0-78.3%) higher in 2007 and 53.3% (95% CI = 33.0-76.7%) higher in 2008. The overall rate of presentations involving any paracetamol-containing drug was 16.4% lower in 2006-2008 than in 2003-2005.

**Table 2:** Use of analgesics in intentional drug overdose presentations to hospital in Ireland before and after the withdrawal of distalgesic

Drug involved in presentations	**2003-5**^1^	**2006-8**^1^	**Change**^1^	**Relative change**^2^	**(95% CI)**^2^
Distalgesic	10.0	1.6	-8.4	-83.5%^3^	(-85.7, -81.0%)
Other prescription compound analgesics	4.2	5.9	+1.7	+43.5%^3^	(+28.6, +60.1%)
Solpadeine	6.3	6.5	+0.2	+2.6%	(-6.7, +12.8%)
Any paracetamol	61.3	51.5	-9.8	-16.4%^3^	(-19.0, -13.7%)

^1 ^Age-standardised rate per 100,000

^2 ^Based on the incidence rate ratio after adjustment for age

^3 ^p < 0.001

**Figure 4:**
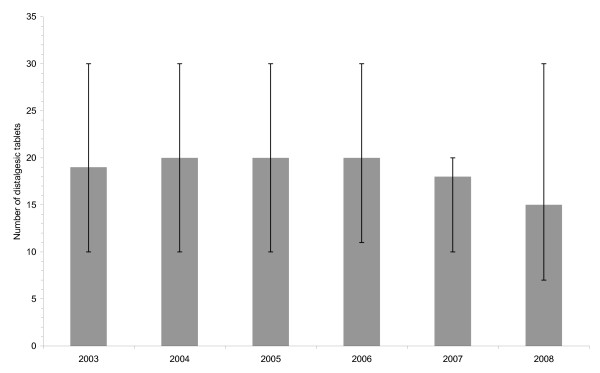
**Median number of distalgesic tablets taken in intentional drug overdose presentations to hospital in Ireland, 2003-2008**. Note: Error bars represent the interquartile range.

The median number of distalgesic tablets taken in IDO presentations involving the drug was 20 (Interquartlie (IQ) range = 10-30), with men, on average, taking more distalgesic tablets in IDO acts than women (Mann-Whitney U = 117,207, p < 0.001; Male median (IQ range) = 20 (12-36); Female median (IQ range) = 18 (10-28)). It can be seen from Figure 4 that the number of distalgesic tablets taken in IDO presentations was relatively constant in 2003-2006 and decreased slightly in 2007 and 2008. However, the yearly variation in the number of distalgesic tablets taken was not statistically significant (Kruskal-Wallis test chi-square = 4.91, df = 5, p = 0.427).

## Discussion

The withdrawal of distalgesic from the Irish market in January 2006 resulted in an immediate reduction in sales to retail pharmacies. The rate of intentional drug overdose (IDO) presentations to hospital involving distalgesic in 2006-2008 was 84% lower than in the three years pre-withdrawal. The magnitude of the impact of the withdrawal was greater than the effects of the withdrawal of distalgesic (co-proxamol) on drug-related suicide mortality in Scotland [7] and in England and Wales [5] where the withdrawal was phased over the three years 2005-2007. Scottish poisoning deaths involving co-proxamol as the sole agent were 50% lower in 2005-2006 than in 2000-2004 [7]. In England and Wales, the three withdrawal years saw a decrease of 59% in the prescribing of co-proxamol and a 62% decrease in suicide deaths compared to 1998-2004 [5]. In Sweden, restrictions on the prescribing of drugs containing dextropropoxyphene were implemented in 2000-2002 and these were associated with a 66% decrease in sales, a 62% decrease in the number of dextropropoxyphene poisoning deaths and a 28% decrease in the number of dextropropoxyphene inquiries to the Swedish Poisons Information Centre [6].

In Ireland, the 48% jump in sales of the other prescription compound analgesics (Kapake, Paramol, Solpadol, Syndol and Tylex) between 2005 and 2006 was likely to have been due to doctors prescribing these drugs as alternatives to the withdrawn distalgesic. The 11% increase in sales of solpadeine and 22% increase in sales of other paracetamol-containing medicines may have been partially related to the withdrawal of distalgesic. However, these increases were not greatly out of line with the annual increases in the sales of these drugs in the years before distalgesic was withdrawn. There was also evidence of a degree of substitution in respect of IDO presentations to hospital. The increased availability of the other prescription compound analgesics resulted in a significant increase in the rate of IDO presentations to hospital involving these drugs. However, the magnitude of the decrease in distalgesic-related IDO presentations was about five times greater than the magnitude of the increase in IDO presentations involving the other prescription compound analgesics. Recent UK studies of the impact of distalgesic (co-proxamol) withdrawal on suicide mortality found little or no evidence of substitution [5,7]. The total rate of IDO presentations to hospital and the rate of paracetamol-related presentations were decreasing in the three years before the withdrawal of distalgesic and continued to do so until 2008.

We found evidence of a time lag in the effect of the withdrawal of distalgesic on IDO presentations. Sales of the drug to retail pharmacies decreased by 99% in 2006 whereas the rate of distalgesic-related IDO presentations was 72%, 84% and 94% lower in 2006, 2007 and 2008, respectively. The evidence of a reduction in the number of distalgesic tablets taken in overdose acts was very limited and only seemed apparent in 2008 and even then half of those presentations involved more than 15 tablets. These findings may reflect the availability of distalgesic from already-filled prescriptions and a gradual reduction in household stocks. The Dispose of Unused Medicines Properly (DUMP) campaign is a separate initiative also aimed at restricting access to means of suicidal behaviour http://www.imt.ie/opinion/2009/05/dumping_drugs_saves_lives.html. Carried out in a number of areas across Ireland, the DUMP campaign called on the general public to bring medicines that were out-of-date or no longer in use to participating pharmacies where they were collected and subsequently destroyed. The campaign may have accelerated the disposal of distalgesic from the homes of patients. However, it may have more effective had the DUMP campaign been adopted nationally and promoted in conjunction with the withdrawal of distalgesic. In relation to future preventive initiatives, there is a need for greater awareness and promotion of existing initiatives that have the potential to be complimentary.

The study had a number of strengths and limitations. We were able to examine data relating to IDO presentations to the vast majority, though not all, of the hospitals in Ireland. In line with recommendations for the evaluation of initiatives restricting access to means of suicidal behaviour [12], we examined data from the three years before and the three years after the withdrawal of distalgesic. We examined sales to retail pharmacy data to assess changes in the availability of the relevant drugs. However, we did not include sales to hospital pharmacies and therefore our figures underestimate total sales somewhat. Changes in presentation rates and the number of tablets taken were examined for IDO presentations involving distalgesic and other relevant drugs. Substitution to other methods of self-poisoning or self harm was not examined. Legislation restricting pack-sizes of paracetamol-only drugs was implemented during the study period [13] which may explain the decrease in the rate of paracetamol-related IDO presentations observed in the study period. The study was confined to IDO presentations to hospital emergency departments and therefore excluded untreated acts and those managed by general practitioners. It would also have been of interest to have examined suicide data. However, at the time of the study, Irish suicide data were available only up to 2006 and the data could not distinguish deaths involving distalgesic from those involving other analgesics.

## Conclusions

The withdrawal of distalgesic from the Irish market resulted in a marked reduction in the rate of IDO presentations to hospital involving the drug. The smaller increase in IDO presentations involving other prescription compound analgesics constituted evidence of some substitution. However, the withdrawal of distalgesic in Ireland can be considered a positive measure in the prevention of non-fatal suicidal behaviour, which is likely also to have an effect on suicide.

## Competing interests

The authors declare that they have no competing interests.

## Authors' contributions

PC and EA designed the study with contributions from UR. PC, EA and UR drafted the manuscript. PC and UR carried out the data analyses. HSK and IJP were involved in the conception of the Registry upon which the study was based and contributed to revisions of the manuscript. All authors read and approved the final manuscript.

## Pre-publication history

The pre-publication history for this paper can be accessed here:

http://www.biomedcentral.com/1472-6904/10/6/prepub
